# Mitochondrial Oxidation of the Cytoplasmic Reducing Equivalents at the Onset of Oxidant Stress in the Isoproterenol-Induced Rat Myocardial Infarction

**DOI:** 10.3390/antiox10091444

**Published:** 2021-09-11

**Authors:** Olivia Vázquez-Martínez, Mauricio Díaz-Muñoz, Fernando López-Barrera, Rolando Hernández-Muñoz

**Affiliations:** 1 Departamento de Neurobiología Celular y Molecular, Instituto de Neurobiología, Universidad Nacional Autónoma de México (UNAM), Campus UNAM-Juriquilla, Querétaro 76230, Mexico; ovazquez@comunidad.unam.mx (O.V.-M.); mdiaz@comunidad.unam.mx (M.D.-M.); lopezbfe@unam.mx (F.L.-B.); 2 Departamento de Biología Celular y Desarrollo, Instituto de Fisiología Celular, Universidad Nacional Autónoma de México (UNAM), Ave. Universidad # 3000, Coyoacán 04510, Mexico

**Keywords:** cardiac infarction, isoproterenol, malate-aspartate shuttle, oxidative stress, sequential alterations

## Abstract

We have developed and characterized a model of isoproterenol (ISO)-induced myocardial necrosis, identifying three stages of cardiac damage: a pre-infarction (0–12 h), infarction (24 h), and post-infarction period (48–96 h). Using this model, we have previously found alterations in calcium homeostasis and their relationship with oxidant stress in mitochondria, which showed deficient oxygen consumption and coupled ATP synthesis. Therefore, the present study was aimed at assessing the mitochondrial ability to transport and oxidize cytoplasmic reducing equivalents (NADH), correlating the kinetic parameters of the malate-aspartate shuttle, oxidant stress, and mitochondrial functionality. Our results showed only discreet effects during the cardiotoxic ISO action on the endogenous malate-aspartate shuttle activity, suggesting that endogenous mitochondrial NADH oxidation capacity (Nohl dehydrogenase) was not affected by the cellular stress. On the contrary, the reconstituted system showed significant enhancement in maximal capacity of the malate-aspartate shuttle activity only at later times (post-infarction period), probably as a compensatory part of cardiomyocytes’ response to the metabolic and functional consequences of the infarcted tissue. Therefore, these findings support the notion that heart damage associated with myocardial infarction suffers a set of sequential biochemical and metabolic modifications within cardiomyocytes, where mitochondrial activity, controlling the redox state, could play a relevant role.

## 1. Introduction

Tissues and organs with high oxidative performance, such as the myocardium, are highly dependent on adequate mitochondrial activity. Sufficient ATP production requires a coordinated flux of metabolites from the cytosol to within the mitochondrion to contribute reducing equivalents, like nicotinamide adenine dinucleotide hydrogen (NADH) and flavin adenine dinucleotide (FADH_2_), to supply the oxidative phosphorylation [1]. In conditions of enough O_2_ availability and full oxidative mitochondrial capacity, the NADH produced by the cytosolic glycolytic pathway can be used by mitochondria only if the shuttle systems are functional. These biochemical pathways involve the reduction of the substrate in the cytosol that are permeable to the mitochondrial inner membrane. These substrates are then oxidized by the mitochondrion to yield a substrate that can return to the cytosol for a new reduction. In the heart, the principal system is the malate-aspartate shuttle (MAS) since the glycerol-phosphate shuttle is almost undetectable [2].

Coronary heart diseases are prevalent clinical emergencies in western societies and the most relevant pathologies promoting the highest morbidity and mortality. An inadequate supply of oxygen associated with atherosclerosis causes coronary occlusion, myocardial hypoxia, and eventually infarction [3]. Because this pathological condition is crucial for global health, it has been imperative for the biomedical research community to implement experimental models to understand the disease and explore potential therapies. Preclinical animal models are broadly divided into ex vivo and in vivo models. Ex vivo models are further divided into the Langendroff heart model and isolated cardiac cells. In vivo models are sub-classified into small and large animal models. Among the small animal models, the left anterior descending artery ligation (LADAL), genetically modified rodents, and the chemical-induced models are the most employed [4].

Acute treatment with isoproterenol (ISO) is one of the most popular experimental models to generate myocardial infarction. This chemical process has been used successfully in rodents revealing histological injuries in subendocardial regions, localized in the apical cardiac zone. In the first hours, the heart damage is accompanied by oxidative stress and inflammatory response with the onset of infarction 24 h after drug administration [5]. The infarcted animals usually survive, thus, it is possible to study, after the second day of ISO treatment, cellular mechanisms related to cardiac recovery and remodeling [6,7].

The present study aimed to characterize the MAS activity in the hearts of male Wistar rats subjected to treatment with a single dose of ISO. The experiments distinguished the endogenous and the reconstituted MAS activity and were performed at three different stages of the cardiac damage: (1) early phase before 24 h (in our protocol, 3 h and 6 h after ISO administration); (2) myocardial infarction (at 24 h); (3) remodeling phase following the infarcted crisis (in our protocol, 48 h and 96 h after ISO administration). For a better biochemical context, our experiments included a characterization of the cardiac pro-oxidant status and mitochondrial performance determination.

## 2. Materials and Methods

### 2.1. Substrates and Inhibitors 

Enzymes such as malate dehydrogenase (MDH; EC 1.1.1.37) and aspartate aminotransferase (AST; EC 2.6.1.2), coenzymes, 2-oxoglutarate, aspartic and glutamic acids, as well as the ethoxyformic anhydride (EFA; an inhibitor of respiratory components located upstream of the mitochondrial respiratory chain) were obtained from Sigma-Aldrich Chemical Co. (Waltham, MA, USA). The fluorescence probe 2′,7′-dichlorodihydrofluorescein di-acetate (H2DCF-DA), used to signal reactive oxygen species (ROS), was obtained from Molecular Probes, Co, (Eugene, OR, USA). All other reagents were purchased from Merck (CDMX, Mexico).

### 2.2. Myocardial Infarction Model

Male Wistar rats (*n* = 40), weighing 250–280 g of body weight (b.w.) and fed ad-libitum, were injected subcutaneously (s.c.) with (−)-isoproterenol hydrochloride (ISO) at a dose of 70 mg/kg b.w, between 09:00 and 10:00 h (*n* = 25), whereas control rats (*n* = 15) received an equivalent volume of saline solution. Animals were euthanized after 3 and 6 h (pre-infarction period), 24 h (infarction), and 48 h and 96 h (post-infarction period) of ISO administration [6]. Our research was approved by the Animal Experiments Institutional Ethics Committee (Protocol code = CICUAL RHM134-18), according to the Federal Regulations for Animal Experimentation (Ministry of Agriculture, SAGARPA, Mexico).

### 2.3. Cellular Fractionation and Isolation of Heart Mitochondria 

The heart, finely minced, was homogenized in a medium previously described [7] consisting of 180 mmol/L KCl, 10 mmol/L EDTA, and 0.5% free fatty acids (FFA)-free bovine albumin (pH 7.2). For mitochondria isolation, the whole homogenate was centrifuged at 1500× *g* for 10 min; thereafter, the supernatant was filtered through a cheesecloth and spun twice at 8500× *g* for 10 min [8]. The final mitochondrial pellet was additionally washed 3 times and suspended in the same solution (180 mmol/L KCl and 0.5% FFA-free bovine). 

### 2.4. Oxygen Uptake by Isolated Mitochondria 

Mitochondrial respiration and phosphorylation were recorded polarographically with a Clark-type oxygen electrode in 3 mL of a medium containing 250 mmol/L sucrose, 0.5 mmol/L EDTA, and 3 mmol/L potassium phosphate buffer (pH 7.4). Glutamate and malate (5 and 1 mmol/L, respectively) were used as substrates for site I at state 4. Mitochondrial state 3 was initiated by adding ADP (250 µmol/L final concentration). 

### 2.5. Free Radical Assessment and Protein Oxidation

The ROS levels in mitochondrial preparations were estimated through the method described by Viarengo et al. [9], using the fluorescence signal generated by ROS reacting with 2′,7′-dichlorodihydrofluofluorescein di-acetate (H2DCF-DA, Molecular Probes). In the case of the protein-attached carbonyl groups, the denaturalized proteins with trichloroacetic acid were stained with 2,4-dinitrophenylhydrazine and further precipitated with 6 mol/L guanidine dissolved in 20 mmol/L KH_2_PO_4_, measured at 375 nm and finally calculated by its absorption coefficient, according to Levine et al. [10].

### 2.6. Endogenous NADH Oxidation and Reconstitution of the MAS in Heart Mitochondria

Endogenous NADH oxidation by intact mitochondria, assumed to be performed by a heart mitochondrial NADH dehydrogenase described by Nohl [11], was assayed in a medium containing 130 mmol/L KCl, 20 mmol/L KH_2_PO_4_, 20 mmol/L Trisma-HCl, 5 mmol/L MgCl_2_ phosphate buffer (pH 7.2). The reaction was initiated with the addition of 5 mmol/L NADH and its oxidation was monitored spectrophotometrically at 340 nm [12]. 

**Figure 1:**
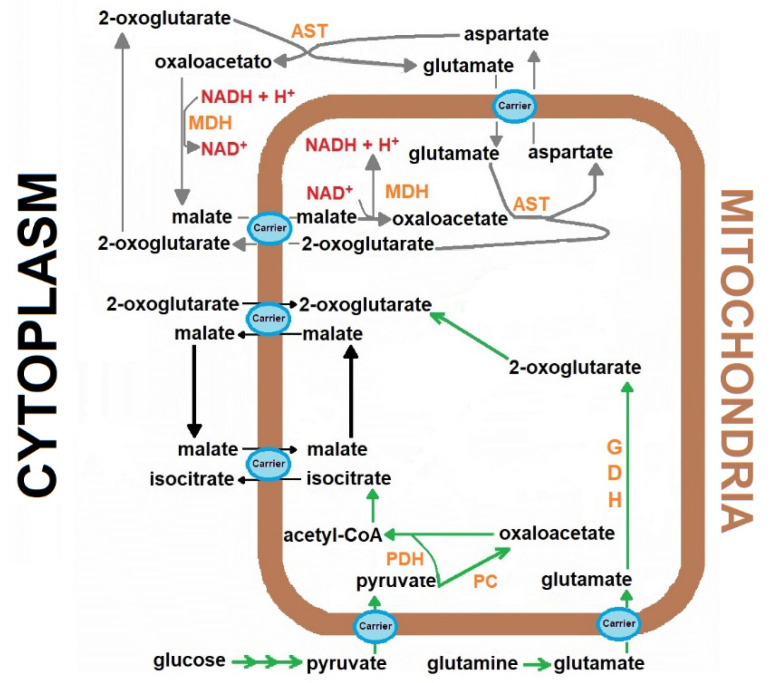
The role of the Malate-aspartate shuttle in the cardiomyocytes metabolism. The scheme represents the transport of the reducing equivalents from the cytoplasm to mitochondria in the heart, through mobilization of metabolites participating in the malate-aspartate shuttle. Abbreviations (enzymes): aspartate aminotransferase (AST), Aspartate aminotransferase, GDH, Glutamic dehydrogenase, malate dehydrogenase (MDH), malate dehydrogenase, PC, Pyruvate carboxylase, PDH, Pyruvate dehydrogenase complex.

The reconstitution experiments were carried out with the addition of the “missing” cytosolic components of the MAS [13], keeping the mitochondrial production of 2-oxoglutarate as the limiting factor for NADH oxidation for the named “reconstituted MAS” (Figure 1). Therefore, the following additions were made: 2 mmol/L aspartate, 5 mmol/L glutamate, 1 mmol/L malate, and 2 and 4 IUnits/5 mL, for MDH and AST, respectively [13]. The shuttle activity was followed through NADH disappearance monitored spectrophotometrically at 340 nm, after the addition of the coenzyme in a range of 5 to 100 µmol/L. 

### 2.7. Preparation of Heart Mitochondrial Particles (NADH Oxidizing Enzymes)

To evaluate the maximal capacity of heart mitochondria to oxidize NADH in the absence of membrane barriers, we prepared sealed particles from rat heart mitochondria. For this, NADH-oxidizing enzyme units were obtained after sonication of heart mitochondria suspended in the ice-cooled isolation buffer, described above. The suspension was sonicated 5 times in a Corex tube for 20 s at 25 W at 15 s intervals. The sonicated suspension was sequentially centrifuged at 9600× *g* for 10 min, and the supernatant at 90,000× *g* for 30 min. Finally, the pellet was sedimented at 105,000 g for 1 h, repeating twice the last step, each time for 30 min; the final pellet was suspended in a solution containing 10 mmol/L TRIS-sodium acetate, and 250 mmol/L sucrose [11]. 

### 2.8. Statistical Analysis

Data are presented as mean ± standard error (SE). The significance of the differences was assessed by Student’s *t*-test. Comparisons among groups were evaluated using one-way analysis of variance (ANOVA) and Bonferroni’s *t*-test with a significance level of *p* < 0.05.

## 3. Results

### 3.1. The Experimental Model of ISO-Induced Myocardial Infarction in Rats 

Using a single administration of ISO, infarct-like damage in the apex region of the left ventricle developed, occurring 12–24 h after ISO administration to rats. The lesion was defined by histological criteria, continuous telemetric electrocardiogram (ECG) recordings, and the increase in serum marker enzymes, specific for myocardial damage. Shortly after ISO treatment, there was an increase in heart rate and a lowering of blood pressure, resulting possibly in functional ischemia (pre-infarction period). Ultrastructural changes and mitochondrial swelling were evident and functional alterations in isolated mitochondria, such as decreases in oxygen consumption, respiratory quotient, ATP synthesis, and membrane potential, were noticed starting at 6 h after ISO administration (pre-infarction period) and lasted until 72 h later (post-infarction period). Mitochondrial proteins decreased after 3 h of treatment, reaching almost a 50% diminution, which was maintained during the whole study. An energy imbalance, reflected by a decrease in energy charge and the creatine phosphate/creatine ratio, was observed at the pre-infarct stage, and also ATP and total adenine nucleotides diminished clearly at this period, reaching a maximum at the onset of infarction and were accompanied by damage to the myocardial function, drastically decreasing left ventricular pressure and shortening the atrioventricular interval (22 h to 25 h after ISO treatment). During post-infarction, a partial recovery of energy charge, creatine phosphate/creatine ratio, membrane potential, and myocardial function occurred, but not of mitochondrial oxygen consumption, rate of ATP synthesis, total adenine nucleotides, or mitochondrial proteins [6,7]. Interesting correlations of the sequential changes in the heart and mitochondrial functions with energy metabolism were obtained at different stages of the isoproterenol-induced cardiotoxicity. 

### 3.2. Parameters of Oxidant Stress and Mitochondrial Substrate Oxidation 

**Figure 2:**
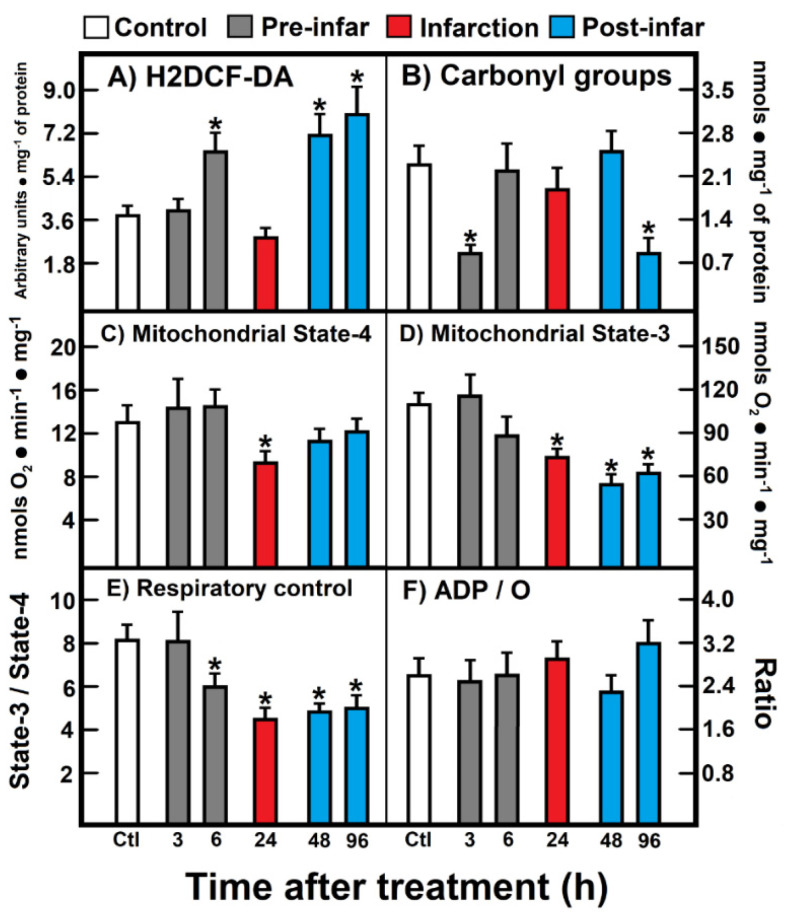
(**A**) 2′,7′-Dichlorofluorescin diacetate (H2DCF-DA); (**B**) Carbonyl groups; (**C**) Mitochondrial State-4; (**D**) Mitochondrial State-3; (**E**) Respiratory control; (**F**) ADP/O. Parameters of oxidant stress and mitochondrial substrate oxidation. Results are expressed as mean ± S.E. of three control animals and five rats treated with ISO per experimental point. Symbols (colors) for the experimental groups at the top of the Figure. Statistical significance: * *p* < 0.05 against the control group.

The level of ROS reacting with H2DCF-DA increased during the pre-infarction period (6 h after ISO treatment), returned to the control level (24 h), and increased again at the post-infarction period (48 h to 96 h post-ISO treatment) (Figure 2A). In contrast, a significant decrease of protein carbonyl groups (oxidized proteins) was noted early at 3 h after ISO treatment, which normalized, thereafter, at the infarction and early post-infarction period (24 h to 48 h after ISO treatment), to be further and unexpectedly diminished at the late post-infarction period (Figure 2B). Isolated heart mitochondria obtained from animals treated with ISO showed significant alterations in the oxidation of substrates like glutamate-malate (Figure 2). Under non-phosphorylating conditions (state 4), a significant diminished mitochondrial respiration was recorded during the infarction period (Figure 2C), which also correlated with a lower response in phosphorylating ADP (state 3) at 24 h after ISO treatment (Figure 2D); however, this diminished mitochondrial state 3 found in animals treated with ISO also persisted at the post-infarction period (Figure 2D). As a consequence, the respiratory control, represented as the state 3/state 4 ratio, was lowered starting the late pre-infarction period (6 h), the infarction, as well as along the whole post-infarction period (Figure 2E). Despite these evident alterations in the heart mitochondrial capacity to oxidize site I substrates, during the ISO-induced myocardial infarction, ADP/O was not significantly affected by the treatment (Figure 2F).

### 3.3. Kinetics of Endogenous Mitochondrial NADH Oxidation and the Reconstitution of the MAS

**Figure 3:**
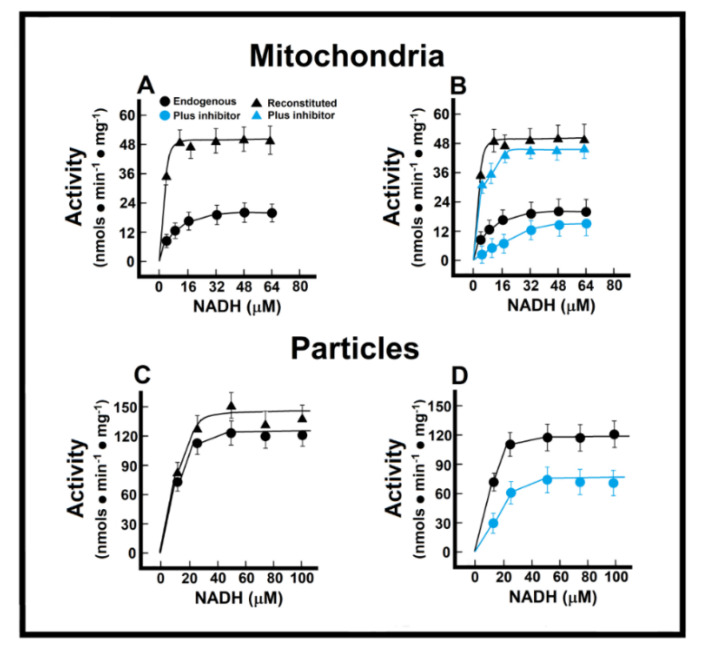
Mitochondrial NADH oxidation without (Panel (**A**)) and with the inhibitor EFA (Panel (**B**)); NADH oxidation by mitochondrial particles without (Panel (**C**)) and with the inhibitor EFA (Panel (**D**)). Kinetics of endogenous mitochondrial NADH oxidation and the reconstitution of the malate-aspartate shuttle. Results are expressed as mean ± S.E. of three control animals and five rats treated with ISO per experimental point of the kinetics of the endogenous or reconstituted malate-aspartate shuttle in the absence or the presence of the inhibitor, ethoxyformic anhydride (EFA). Symbols for the experimental groups at the top of panel (**A**).

In the isolated intact mitochondria, Michaelis–Menten (M-M)-type kinetics were calculated for the endogenous (“Nohl” NADH dehydrogenase) in control preparations (Figure 3). In control preparations, and applying M-M kinetics, we obtained an apparent Km of 8.1 ± 0.9 µmol/L, and a Vmax of 21.0 ± 1.3 nmol·min^−1^·mg^−1^ of protein (Figure 3A). In the healthy adult myocardium, the MAS becomes the main pathway for the transportation of redox products from glycolysis in the cytosol into the mitochondrial matrix, by overpassing the impermeable inner mitochondrial membrane [14]. Therefore, we measured a “reconstituted” MAS pathway, as described in the Methods, to evaluate the functionality of oxidizing cytoplasmic reducing equivalents (Figure 3A). With this approach, control mitochondrial preparations showed a much lower apparent Km (Km of 1.4 ± 0.4 µmol/L) when NADH oxidation also depended on its indirect transfer through the malate shuttle, which was also accompanied by an increase of more than double the apparent Vmax for NADH (51.4 ± 3.3 nmol·min^−1^·mg^−1^ of protein; Figure 3A). The EFA is considered to inhibit selectively NADH-ubiquinone oxidoreductase of complex I [15], without affecting oxidation of exogenous NADH; therefore, with the use of EFA, we were able to distinguish the participation of the external (Nohl) NADH dehydrogenase in the control rat heart. The addition of EFA did not affect significantly the Vmax of endogenous NADH oxidation (26.5 ± 2.3 nmol·min^−1^·mg^−1^ vs. 21.0 ± 1.3 nmol·min^−1^·mg^−1^ of protein, in controls; Figure 3B), but induced a competitive inhibition of the oxidation of endogenous NADH (Km of 59.1 ± 4.5 µmol/L against 8.1 ± 0.9 µmol/L, in controls; Figure 3B).

Preparation of “sub-mitochondrial” particles led us to assess the capacity of heart enzymes to serve as an efficient redox shuttle from cytosolic NADH sources to the respiratory chain and vice versa. The low Km value suggested a high sensitivity of the oxidative enzyme activity towards changes in the cellular NADH/NAD^+^ ratio, without transport and permeability of substrates interfering factors. Under these conditions, we obtained a Km for NADH oxidation of 2.8 ± 0.3 µmol/L, and a Vmax of 129.0 ± 14.3 nmol·min^−1^·mg^−1^ of protein (Figure 3C). As expected, the addition of the missing components of the MAS to the particle’s preparation did not significantly change the kinetic parameters of ethanol oxidation (endogenous vs. reconstituted; Figure 3C). Here, EFA significantly diminished the Vmax of the particles for NADH oxidation (88 ± 11 nmol·min^−1^·mg^−1^ against 129.0 ± 14.3 nmol·min^−1^·mg^−1^ of protein, in controls; Figure 3D), which indicated that activity of the Nohl NADH dehydrogenase is linked to the NADH-ubiquinone oxidoreductase of complex I of the mitochondrial respiratory chain.

### 3.4. Changes of Endogenous NADH Oxidation and the MAS Activity during Installation of Myocardial Infarction

**Figure 4:**
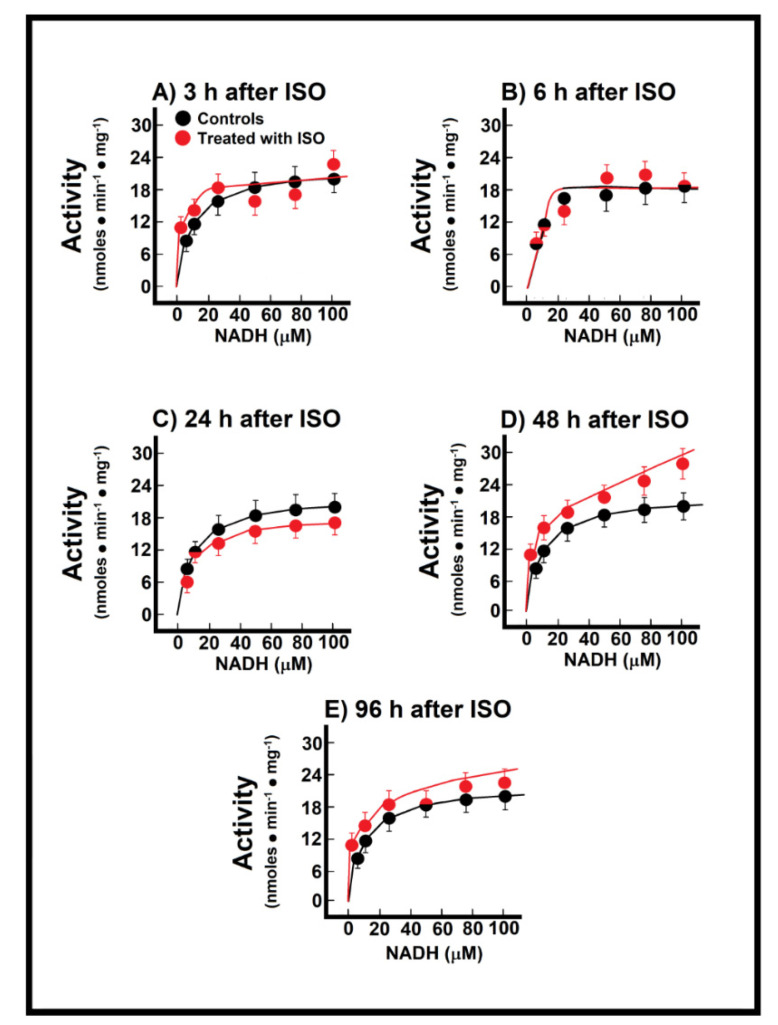
Temporal course of endogenous NADH oxidation at 3 h (Panel (**A**)), 12 h (Panel (**B**)), 24 h (Panel (**C**)), 48 h (Panel (**D**)) and 96 h (Panel (**E**)) after ISO treatment. Changes of endogenous NADH oxidation during induction of myocardial infarction. Results are expressed as mean ± S.E. of three control animals and five rats treated with ISO per experimental point of the kinetics of the reconstituted malate-aspartate shuttle (MAS). Symbols for the experimental groups at the top of panel (**A**). Temporal course of endogenous NADH oxidation at 3 h (Panel (**A**)), 12 h (Panel (**B**)), 24 h (Panel (**C**)), 48 h (Panel (**D**)) and 96 h (Panel (**E**)) after ISO treatment.

During the named pre-infarction period (3 h to 6 h after ISO treatment), there were no significant changes in the M-M kinetics parameters of the endogenous NADH oxidation in the ISO-treated animals (Figure 4A,B). At the onset of infarction (24 h post-ISO), there was only a decreasing trend of both the apparent Km and Vmax for endogenous NADH oxidation when compared with the controls (Figure 4C), but there was a significant increase in the Vmax value (25.5 ± 1.5 nmol·min^−1^·mg^−1^ vs. 21.0 ± 1.3 nmol·min^−1^·mg^−1^ of protein, in controls, *p* < 0.05; Figure 4D) at the early post-infarction period (48 h), which was “normalized” at the later time (96 h after ISO treatment; Figure 4E). Therefore, data indicated that the endogenous oxidation of NADH with the participation of Nohl dehydrogenase was not significantly affected at the onset of myocardial infarction.

**Figure 5:**
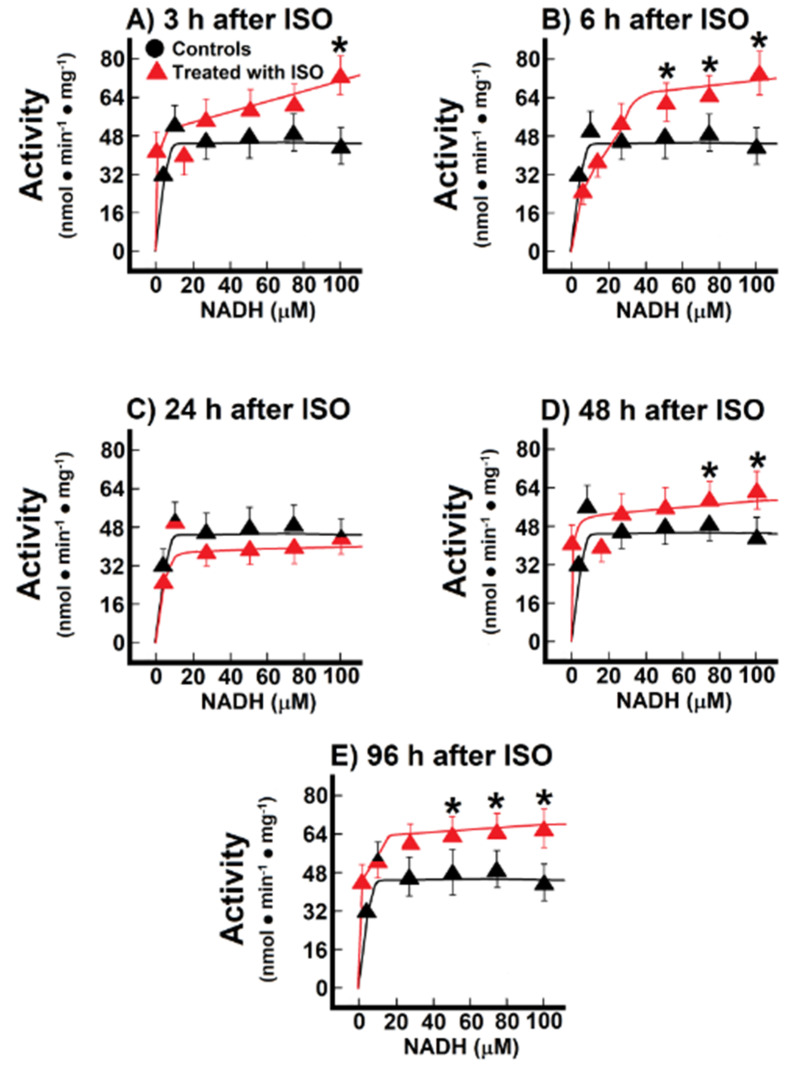
Temporal course of malate-aspartate shuttle at 3 h (Panel (**A**)), 12 h (Panel (**B**)), 24 h (Panel (**C**)), 48 h (Panel (**D**)) and 96 h (Panel (**E**)) after ISO treatment. Changes of the activity of the malate-aspartate shuttle during induction of myocardial infarction. Results are expressed as mean ± S.E. of three control animals and five rats treated with ISO per experimental point of the kinetics of the endogenous NADH oxidation. Symbols for the experimental groups at the top of panel (**A**).

When we recorded mitochondrial NADH oxidation after reconstituting the MAS, we found significant changes in these parameters (Figure 5). During the pre-infarction period, the Vmax for the shuttle was significantly increased at 3 h (71.0 ± 3.2 nmol·min^−1^·mg^−1^ vs. 47.5 ± 2.3 nmol·min^−1^·mg^−1^ of protein, in controls, *p* < 0.01; Figure 5A), as well as at 6 h after ISO treatment (77.1 ± 4.8 nmol·min^−1^·mg^−1^ against 47.5 ± 2.3 nmol·min^−1^·mg^−1^ of protein, in controls, *p* < 0.01; Figure 5B). Moreover, the Km was also significantly increased after ISO treatment, either at 3 h (14.8 ± 1.6 µmol/L vs. 1.4 ± 0.4 µmol/L, in controls, *p* < 0.01; Figure 5A), or at 6 post-ISO (9 ± 1 µmol/L vs. 1.4 ± 0.4 µmol/L, in controls, *p* < 0.05; Figure 5B). Interestingly, at the occurrence of myocardial infarction (24 h) there were no significant modifications of the kinetic parameters for the MAS (Figure 5C), but, in the post-infarction period, there was a significant increase of the Vmax of the MAS activity. The Vmax increased at 48 h (62.5 ± 3.0 nmol·min^−1^·mg^−1^ vs. 47.5 ± 2.3 nmol·min^−1^·mg^−1^ of protein, in controls, *p* < 0.05; Figure 5D), as well as at 96 h after ISO treatment (66.9 ± 2.7 nmol·min^−1^·mg^−1^ vs. 47.5 ± 2.3 nmol·min^−1^·mg^−1^ of protein, in controls, *p* < 0.01; Figure 5E). These changes seem to occur as a compensatory mechanism for the loss of the functionally mitochondrial mass in the severely damaged cardiac tissue [6]. Indeed, the inverse correlation between the increased MAS activity with the lower capacity of coupling phosphorylation, seen in isolated mitochondria during the post-infarction period, gives further support that changes in the MAS activity are functioning as a putative compensatory mechanism (Figure 2 and Figure 5)

## 4. Discussion

Cardiotoxicity related to isoproterenol treatment has been used in multiple studies to evaluate protective actions of a variety of pharmacological and nutritional remedies, for review see [16]. However, very few reports exist regarding the biochemical alteration that characterizes the progressive damage suffered by the cardiac organ induced by the β-adrenergic agonist.

In the past, our group used the ISO-induced cardiac apical infarction as an experimental model to explore metabolic and cellular adaptations in three different stages of heart damage: pre-infarction, infarction, and post-infarction. Particular sequential alterations, as well as adaptations, were shown in the cardiac energy status and intracellular calcium handling [6,7]. Each period of the cardiac damage involved a very specific set of changes, supporting the assumption that a better understanding of the ISO-associated heart injury must consider the progressive installation of intracellular events in the cardiac muscle along with the three phases: first, the deleterious consequences of the enhanced chronotropic and inotropic responses; second, the adaptations to the cellular stress that culminates with the heart apical infarction; third, the remodeling process that promotes structural and functional adjustments in the cardiac organ after recovery of the tissue [6].

In this report and using a similar experimental protocol, we extended our previous metabolic studies to characterize the mitochondrial capacity to consume oxygen, the pro-oxidant status, and the activity of the malate-aspartate shuttle. Our experiments sampled the whole heart to perform the metabolic determinations; hence, the findings represent global cellular alterations associated with the apical necrosis that characterizes this pathological model.

Despite knowing that heart damage by ISO is accompanied by enhanced oxidative stress, structural instability, mitochondrial loss, and ionic imbalance [17], no reports exist regarding the possible modifications shown by the MAS. PubMed search with isoproterenol and infarction as keywords gave 1649 results, when adding the term malate-aspartate shuttle, the result was null (28 June 2021).

The MAS plays a strategic role in the cytoplasmic redox state by allowing the mitochondrial oxidation of the NADH generated by glycolysis and other reactions, such as phosphoglycerate dehydrogenase, required for the synthesis of important metabolites, for example, serine and glycine [18]. The MAS activity involves the coordinated function of enzymes from cytosol and mitochondria, and the presence of specialized mitochondrial transporters (Figure 1). Overall, the MAS function is energy-dependent and powered by the proton-motive force, mainly in the efflux that allows aspartate to reach the cytosolic milieu [19]. Mitochondrial defects influence the MAS performance and lead to redox imbalance characterized by elevated cytosolic NADH/NAD^+^ ratio with metabolic consequences [20]. This condition is even more important within the heart since alternative NADH shuttle systems, like the glycerol-phosphate cycle, are unimportant in the cardiac organ [2].

Our results showed only discreet effects during the cardiotoxic ISO action on the endogenous cardiac NADH dehydrogenase activity, since only after 2 and 4 days of treatment (in the post-infarction stage), was observed an enhancement and a tendency to increase the maximal NADH oxidation (Figure 4). This result could be explained by a putative elevation in the expression or activity of the NADH oxidation system promoted by the plastic adaptations that occur during the remodeling process post-infarction; more experiments are needed to clarify this point. Indeed, the MAS can be kinetically limited by low concentration of implicated metabolites [21], however, the lack of relevant effects on the endogenous MAS activity during ISO-promoted infarction is suggestive that the mitochondrial NADH oxidation capacity, reported by Nohl [11], was not affected by the cellular stress. Interestingly, the reconstituted system showed significant enhancement in maximal capacity of the MAS activity only at the times before the apical necrotic outcome (at 48 and 96 h after ISO administration). At this phase, the activated MAS could be part of the cardiomyocytes’ response trying to compensate for the consequences of the infarcted tissue: the altered ionic fluxes, the energy compromised, and the pro-oxidant metabolic stress [1] and Figure 2. In line with this finding, it has been reported that the pre-ischemic.

MAS inhibition by pharmacological treatment is capable to offer cardioprotection in a model of 40-min global no-flow ischemia experimental model [22].

## 5. Conclusions

These findings support the notion that heart damage associated with myocardial infarction should be understood as a set of sequential biochemical and metabolic modifications within cardiomyocytes. Even though that each stage around the infarction event is characterized by its own and particular set of alterations (pro-oxidant reactions, ionic imbalance, inflammatory responses), to understand better the global process it is essential to consider a special role for mitochondrial activity and redox state control.

## Data Availability

Data is contained within the article.
